# Inhibition of Poly(ADP-Ribose) Polymerase Enhances Radiochemosensitivity in Cancers Proficient in DNA Double-Strand Break Repair

**DOI:** 10.3390/ijms14023773

**Published:** 2013-02-08

**Authors:** Lauren Shunkwiler, Gina Ferris, Charles Kunos

**Affiliations:** Department of Radiation Oncology, CASE Comprehensive Cancer Center, University Hospitals Case Medical Center and Case Western Reserve School of Medicine, Cleveland, OH 44106, USA; E-Mails: lbs52@case.edu (L.S.); gjf3@case.edu (G.F.)

**Keywords:** poly(ADP-ribose) polymerase, veliparib, cervical cancer, radiation, topotecan

## Abstract

Pharmacologic inhibitors of poly(ADP-ribose) polymerase (PARP) putatively enhance radiation toxicity in cancer cells. Although there is considerable information on the molecular interactions of PARP and BRCA1- and BRCA2-deficient cancers, very little is known of the PARP inhibition effect upon cancers proficient in DNA double-strand break repair after ionizing radiation or after stalled replication forks. In this work, we investigate whether PARP inhibition by ABT-888 (veliparib) augments death-provoking effects of ionizing radiation, or of the topoisomerase I poison topotecan, within uterine cervix cancers cells harboring an unfettered, overactive ribonucleotide reductase facilitating DNA double-strand break repair and contrast these findings with ovarian cancer cells whose regulation of ribonucleotide reductase is relatively intact. Cell lethality of a radiation-ABT-888 combination is radiation and drug dose dependent. Data particularly highlight an enhanced topotecan-ABT-888 cytotoxicity, and corresponds to an increased number of unrepaired DNA double-strand breaks. Overall, our findings support enhanced radiochemotherapy toxicity in cancers proficient in DNA double-strand break repair when PARP is inhibited by ABT-888.

## 1. Introduction

Cell death after ionizing radiation (IR) and topoisomerase I poisons such as topotecan occurs by irreparable nuclear or mitochondrial DNA damage. IR instantaneously generates both single-stranded (SSB) and double-stranded breaks (DSB) in DNA that are repaired by base excision, by homologous recombination, or by nonhomologous end-joining pathways. In contrast, topotecan intercalates between bases in supercoiled DNA, stalls DNA replication forks, prevents relegation of the cleaved DNA strand, and induces DSBs [1]. To accomplish timely repair of damaged DNA, cells depend on deoxyribonucleotides supplied *de novo* by ribonucleotide reductase (RNR) [2]. Cancer cells arising in the uterine cervix have an increased capacity for DNA DSB repair mediated through overactivity of RNR that happens as a result of virally-silenced or mutated p53 [3–6]. Because cervical cancers possess abundant deoxyribonucleotide supply through an uncontrolled RNR, cervical cancers may be exquisitely sensitive to targeted biologic agents that protract and disrupt radiation-related and chemotherapy-related DSB repair.

Eukaryotic poly(adenosine diphosphate (ADP)-ribose) polymerase (PARP) is an enzyme that senses DNA-strand breaks and facilitates their repair [7,8]. Of the 18 nuclear proteins in the PARP superfamily, PARP-1 and PARP-2 are generally recognized as tankyrase enzymes that are primarily involved in base excision DNA repair [9]. Common to all PARP family members, the catalytic site binds nicotinamide adenine dinucleotide (NAD+) and generates a branching scaffold of poly(ADP-ribose) (PAR) polymers that confer a strong, covalently attached negative charge to targeted proteins [10]. PAR polymers may facilitate or accelerate repair process through recruitment of DNA polβ [11], X-ray repair cross-complementing factor 1 (XRCC1, [11,12]), and DNA ligase III [13]. PARP-1 activity is enhanced 500-fold when bound to DNA strand breaks; in the absence of such binding, synthesis of PAR polymers is negligible [7]. In knockout mouse models, 80%–90% of PARP-dependent repair activity is significantly blocked with depletion of PARP-1 [14]. Residual PARP-dependent repair in these mice is due to PARP-2 [15]. Together, this suggests that only PARP-1 and PARP-2 need to be inhibited to impair SSB and DSB repair [16].

ABT-888 (veliparib) is an orally available small molecule, equipotent inhibitor of PARP-1 and PARP-2 [17]. The expression of PARP is higher in tumor cells compared to normal cells [18], and its overexpression is linked to cytotoxic drug resistance and the ability of tumor cells to withstand genotoxic stressors. Two-fold overexpression of PARP-1 protein has been shown in cervical cancers as compared to normal uterine cervix cells [18].

To clarify the radiochemosensitizing impact of PARP inhibition in cervical cancer, we studied the effects of ABT-888 (veliparib) upon IR- and topotecan-related DSB repair. The combination of topotecan and ABT-888 is currently under phase II clinical trial testing in women with persistent or recurrent cervical cancer (Gynecologic Oncology Group protocol #127W), making this study particularly relevant in molecular cancer biology. Our data in particular point to enhanced IR-ABT-888 and topotecan-ABT-888 lethality, supporting the contention that the sensitizing effects of PARP inhibitors relate to unrepaired DSBs at 24 h after DNA damaging insults.

## 2. Results and Discussion

### 2.1. Poly(ADP-Ribose) Polymer Formation after Ionizing Radiation, Topotecan, and ABT-888 Exposure

Build-up of PAR polymer scaffolds has been used as a sensitive measure of cell exposure to IR and as an indicator of repair following DNA damage [11–14,19]. Loss of PARP activity in a cell may lead to an increased number of unrepaired, lethal radiochemotherapy lesions. To first understand the underlying basis of sensitivity resulting from PARP inhibition in a cell, we tested for PAR polymer scaffolds using a validated chemiluminescence assay after treatment with radiation or topotecan to induce DSBs. Given our interest in supporting clinical trial work, we conducted PAR assays in mutational-silenced p53^mut/mut^ C33-a cervical cancer [20], human papillomavirus-silenced p53^+/+^ CaSki cervical cancer [21], and SKOV3 ovarian cancer cells [22]. A key novel concept probed here is the construction and deconstruction kinetics of PAR polymer scaffolds in the backdrop of an unchecked, overactive ribonucleotide reductase enzyme supplying deoxyribonucleotides as building blocks for DNA repair in the uterine cervix cancer cell lines [3,4].

**Figure 1:**
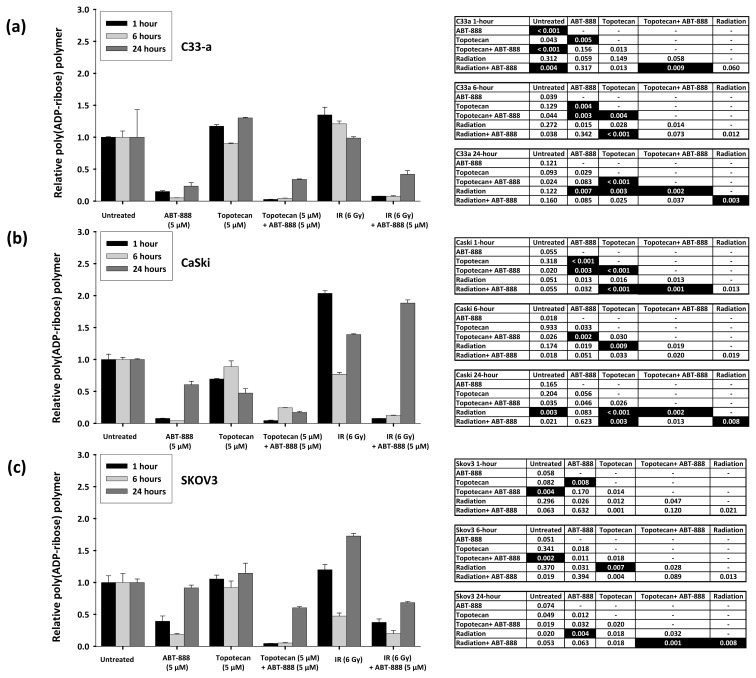
(**a**–**c**) Level of poly(ADP-ribose) polymer after ABT-888 (5 μM), topotecan (5 μM), or ionizing radiation (IR, 6 Gy) alone or after indicated combination in C33-a, CaSki cervical cancer cells or SKOV3 ovarian cancer cells. Means and standard errors are charted. Accompanying tables indicate significant differences in poly(ADP-ribose) polymer (black boxes, *p* < 0.01) relative to untreated cells at the corresponding time point.

IR-mediated DNA damage is repaired typically within a 4-h time span [3]. Thus, we were most interested in any rises in PAR levels within the first 6 h after IR. PAR was found to be elevated at 1 h after IR, and then returned to near baseline 6 h after initial exposure to IR (Figure 1a–c). As it turned out, the 1-h PAR level inversely corresponded to the relative rank of radiation sensitivity, as will be discussed subsequently. Further refinement in 6-h PAR kinetics was complicated by the cytotoxicity of radiation and PARP inhibition.

Because the levels of PAR reflect DSBs generated during uncoiling of DNA, we also examined the levels of PAR in topotecan-treated cells. PAR signal changed modestly 1 h after topotecan treatment as a single agent (Figure 1a–c). As time elapsed after initial topotecan 6-h exposure, PAR levels remained similar.

We were next interested in whether PARP activity could be blocked by ABT-888 under conditions where demand to fix damaged DNA is high. Previous studies have shown that PAR polymer formation can be inhibited by ABT-888 at a clinically-relevant dose of 5 μM [17]. The addition of ABT-888 as a single agent resulted in a dramatic reduction in detected PAR polymer levels at 1 and 6 h after initial drug exposure (Figure 1a–c). By at least the 24 h time point after initial ABT-888 exposure (*i.e.*, 18 h after a 6-hour ABT-888 treatment), there was evidence of bounce-back recovery of PARP enzymatic activity (Figure 1a–c). After IR or after topotecan, PAR levels were low in the co-presence of ABT-888, and again, a signal for enzyme recovery at 24 h was detected (Figure 1a–c). Our studies do not resolve any of the possible molecular means of enzyme recovery, such as post-translational modification or new synthesis of functional enzyme [23,24].

### 2.2. Radiochemosensitivity Is Enhanced by PARP Inhibition in Cells Proficient in DNA Damage Repair

We next set out to evaluate the impact of PARP inhibition upon IR and topotecan cytotoxicity. Cervical cancer cells have been shown to be highly proficient in DNA damage repair due to overactive RNR [3–5]. Whether PARP inhibition sensitizes cells to the effect of IR or topotecan, in the setting of overactive RNR enzyme capable of facilitating DNA damage, has not been fully characterized.

**Figure 2:**
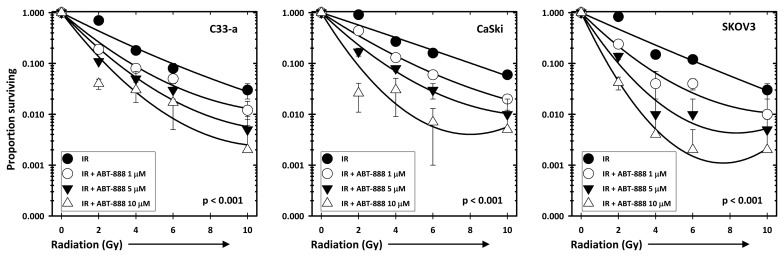
Clonogenic cell survivals after ionizing radiation (IR) and ABT-888 are illustrated for C33-a, CaSki cervical cancer and SKOV3 ovarian cancer cells. Radiation dose and ABT-888 concentration are listed in the legend. A significant dose-dependent, positive radiosensitization effect of ABT-888 is shown (*p* < 0.001). Means and standard errors are shown.

Relative to untreated cells, the mean cytotoxicity of a 2 Gy clinical radiation dose was 9% (SE = 2%) in CaSki cells, 30% (SE = 2%) in C33-a cells, and 17% (SE = 2%) in SKOV3 cells. A clinically-relevant dose of ABT-888 (5 μM) had a minimal cytotoxic effect by itself, with mean cell death of only 4% (SE = 1%) in CaSki cells, 2% (SE = 1%) in C33-a cells, and 6% (SE = 1%) in SKOV3 cells. In contrast, a potentiating effect was evident after topotecan (5 μM) plus PARP inhibition by ABT-888 (5 μM). Gains in cytotoxicity were modest when comparing topotecan alone versus topotecan plus ABT-888 (CaSki: 78% (SE: 1%) *vs.* 93% (SE: 1%), *p* < 0.001; C33-a: 78% (SE: 8%) *vs.* 97% (SE: 1%), *p* < 0.01; SKOV3: 74% (SE: 6%) *vs.* 96% (SE: 2%), *p* < 0.01, respectively). In conventional radiation clonogenic survival assays, ionizing radiation (IR) plus PARP inhibition resulted in greater gains in cell cytotoxicity at each incremental increase in ABT-888 dose relative to IR alone (each, MANOVA *p* < 0.001; Figure 2). Substantial gains in cytotoxicity are observed at the clinical 2 Gy radiation dose, increasing with ABT-888 concentration. At clinically ablative radiation doses (10 Gy), the beneficial toxic effects of PARP inhibition after radiation-induced DNA damage appear muted (Figure 2).

Our cumulative data suggest that PARP inhibition may promote lethal accumulation of DSBs from IR or topotecan, and that these breaks may be more toxic in the absence of overactive DNA damage repair mechanisms. To the latter point, cervical cancer cells lack functional p53 protein, with derailment in p53 activity either by human papillomavirus (CaSki) or by mutation (C33-a) [3]. As a consequence of silenced p53 activity, cells either may have an overactive RNR capable of supplying deoxynucleotides unfettered [3] or be actively engaged in S-phase DNA duplication wherein deoxynucleotide production is high [25]. Both cervical cancer cell lines are afforded an increased opportunity to fix damaged DNA readily because of an elevated deoxynucleotide output from RNR. The SKOV3 ovarian cancer cells do not necessarily have the same deoxynucleotide payoff from RNR, and thus, appear slightly more sensitive to the death-provoking effects of IR and IR plus ABT-888 (Figure 2).

When ABT-888 was used to block PARP and disrupt DNA damage responses relying on PAR scaffolding, cytotoxicity was most pronounced in cells confronted with stalled replication forks. We observed that PARP inhibition modestly increased IR cytotoxicity (Figure 2), but had a more lethal effect in cells treated by topotecan. It is therefore reasonable to conclude that SSBs created by topotecan lead to replication-dependent generation of lethal DSBs, repair of which is hampered by an ABT-888 inhibition of PARP. This effect perhaps would account for the increased cytotoxicity seen after topotecan-ABT-888 treatment. When PARP is inhibited by ABT-888 (Figure 1) but RNR activity is overactive by silencing of p53 or is induced by IR [3], it is expected that more *de novo* deoxynucleotides would be available for faster DNA repair and cytotoxicity would be lessened, as observed (Figures 1 and 2). Our findings are consistent with prior work where PARP inhibition had a greater toxic effect on cells kept under hypoxic conditions [26] or on ATM^−/−^ cells [23]. Survival effects after IR or topotecan with added ABT-888 implicate increased conversion of non-lethal to lethal double-strand breaks as one of the underlying mechanisms of radiochemosensitization by PARP inhibitors, and these phenomena are discussed next.

### 2.3. DNA Double-Strand Break Resolution after Radiation or Topotecan Is Delayed by PARP Inhibition

DSBs are perhaps more lethal if repair is protracted and if the break persists at 24 h [27]. While the mechanism accounting for this remains under study, it is tempting to speculate that close or opposing double nicks in a DNA backbone allow for “free” ends of the DNA to drift apart. Without scaffolding to hold the “free” ends in close proximity, there is an increasing probability that the two ends may not be re-hooked. When IR generates DSBs or topotecan induces stalled replication forks, cells are challenged to fasten DSB free ends back together in a relatively short time period. With this in mind, we first investigated IR-induced γH2AX (H2AX phosphorylation at Ser-139) foci resolution by cytometry in only C33-a cervical cancer cells as a pilot detection test of the γH2AX DNA damage signal. IR-treated cells had minimal delay in resolution of IR-induced γH2AX signal when PARP was not inhibited, but substantial protraction of IR-induced γH2AX signal occurred when PARP was blocked by ABT-888 (Figure S1).

**Figure 3:**
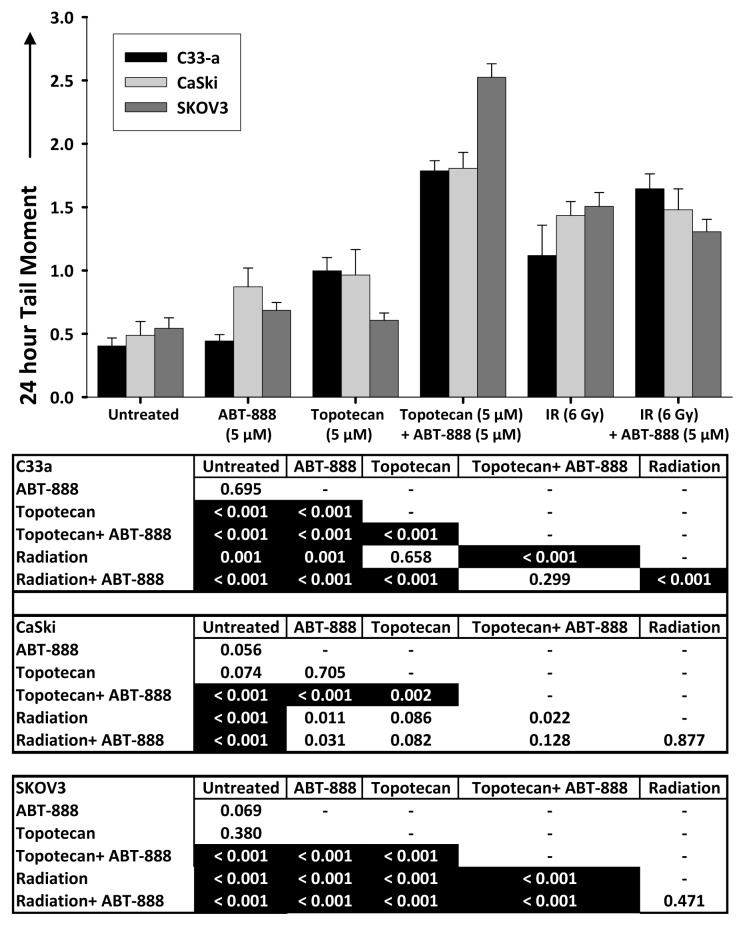
Neutral-condition single cell electrophoresis assay at 24 h after ABT-888 (5 μM), topotecan (5 μM), or ionizing radiation (IR, 6 Gy) alone or after indicated combination in C33-a, CaSki, or SKOV3 cells. Graphed are means and standard errors of 24 h DNA tail moment, defined as the product of the tail length and the total DNA fraction in the tail.

Because we could not resolve whether γH2AX signal was due to “true” IR-related DSBs or simply due to DNA replication forks, as both would emit detectable γH2AX signal, we sought whether PARP inhibition protracted repair of IR-induced or topotecan-induced DNA double-strand breaks by neutral comet assay in the three cell lines. Neutral comet assays done 24 h after ABT-888 exposure alone (*i.e.*, 18 h after a 6-h ABT-888 treatment) showed no increase in DSBs (Figure 3). When compared to untreated cells, IR created significant (*p* < 0.001) numbers of unrepaired DSBs, as evidenced by larger DNA tail moments (Figure 3). But, the addition of ABT-888 to IR did not increase (*p* > 0.01) the number of unrepaired DSBs when compared to IR alone (Figure 3). Topotecan slightly increased DNA tail moments at 24 h post-exposure, suggesting accumulation of unrepaired DSBs (Figure 3). The combination of topotecan and ABT-888 did show significantly larger DNA tail moments suggestive of many retained DSBs (Figure 3). The implications of these data are first that stalled or collapsed replication forks induced by topotecan result in the conversion of SSBs to DSBs that can be detected many hours after they first occur. Such data are consistent with the hypothesis that PARP inhibition protracts DSB repair leading to cytotoxicity.

**Figure 4:**
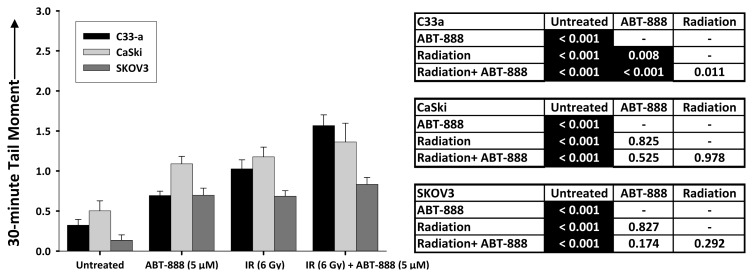
Neutral-condition single cell electrophoresis assay at 30 min after ABT-888 (5 μM) or ionizing radiation (IR, 6 Gy) alone or after an IR-ABT-888 combination in C33-a, CaSki, or SKOV3 cells. Means and standard errors for 30 min DNA tail moment, defined as the product of the tail length and the total DNA fraction in the tail, are provided.

A second implication of these data is that radiosensitization by PARP inhibition may not be mediated at all by interference with repair of DSBs. Nonhomologous end-joining or homologous repair of DSBs occurs quickly in cells; so, PARP inhibition effects observed 24 h after ABT-888 exposure may not be the result of altered DSB repair kinetics. We tested this notion by a neutral single cell electrophoresis assay conducted at 30 min after IR in the three cell lines (Figure 4). Relative to untreated cells, cells treated by ABT-888, by IR and by ABT-888 added to IR all had larger DNA tail moments indicative of increased numbers of DSBs (Figure 4). At the 30 min time point and relative to IR treated cells, adding PARP inhibition by ABT-888 only increased the mean DNA tail moment in the mut-p53 C33-a cervical cancer cells, while the virally-silenced wt-p53 CaSki cervical cancer cells and SKOV3 ovarian cancer cells did not show this effect (Figure 4). This raises the possibility of as yet unrecognized molecular targets of PARP that may be responsible for the radiosensitizing properties of PARP inhibition [23], or commitment to apoptosis [24], or that ABT-888 is more effective in some cells than others (Figure 1).

## 3. Experimental Section

### 3.1. Cell Cultures and Chemicals

Human cervical cancer cells C33-a (human papillomavirus (HPV)-naïve, mutated p53 (codon 273 Arg-Cys), [20]) and CaSki (HPV-16 positive, wt-p53, [21]) and human ovarian cancer cells SKOV-3 [22] were obtained from American Type Culture Collection (Rockville, MD, USA). Cells were cultured at 37 °C in a humidified 5% CO_2_ atmosphere using RPMI 1640 (ThermoFisher Scientific, Waltham, MA, USA) supplemented with 10% fetal bovine serum, 1% l-glutamine and 1% penicillin/streptomycin. Exponentially-growing cells were cultured in experimental plates for 24 h prior to any radiation or drug exposure. Chemicals used were purchased from Sigma (St. Louis, MO, USA) unless otherwise stated.

### 3.2. Radiation and Drug Treatments

To generate DNA double-strand breaks, IR (0, 2, 4 or 8 Gy) was delivered using a ^137^Cs γ-irradiator (JL Shepherd Associates, San Fernando, CA, USA) at a dose rate of 3.23 Gy/min. For radiation-drug treatments, drugs were added to the medium immediately after irradiation. To create stalled replication forks collapsing to DNA double strand breaks, topotecan was used at the clinically-relevant doses of 0–10 μM. ABT-888 (veliparib, NSC #737664) is an investigational agent provided to Case Western Reserve University (Cleveland, OH, USA) under an agreement with the National Cancer Institute Cancer Therapy Evaluation Program (NCI-CTEP, Bethesda, MD, USA) and Abbott Laboratories (Chicago, IL, USA). To inhibit PARP activity at clinically relevant doses, ABT-888 was used at end concentrations of 0–10 μM. For poly(ADP-ribose) enzyme, neutral single cell electrophoresis, and clonogenic survival assays with cell harvest time greater than 6 h, topotecan-containing or ABT-888-containing medium was exchanged for fresh drug-free medium at the 6-h post-exposure time point.

### 3.3. Clonogenic Survival Assays

Cells were plated in triplicate to yield 300 or 3000 cells per 60-mm culture plate. Cells received IR (0, 2, 4 or 8 Gy) or in combination with a 6-h exposure of ABT-888 (0, 1, 5 or 10 μM), a time period selected to mimic *in vivo* ABT-888 pharmacokinetics [17]. Seven days after plating and treatment, surviving colonies (>50 cells) were fixed with 75% ethanol, stained with 0.5% crystal violet, rinsed, dried, and counted. Untreated control cell plating efficiency normalized colony counts.

### 3.4. Poly(ADP-Ribose) Polymerase Activity Assay

Cultures of 2 × 10^6^ cells were plated in 10 cm dishes. For detection of biotinylated poly(ADP-ribose) (PAR) in treated cells, the commercial 96-well Universal PARP assay kit (#4520-096-K, Trevigen^®^ Inc., Gaithersburg, MD, USA) was used according to the manufacturer’s instructions and supplied reagents. Cells were harvested at 1, 6, and 24 h after indicated treatment. After processing, PAR rabbit polyclonal antibody (1:250) was added in 50 μL aliquots followed by goat anti-rabbit IgG antibody conjugated with horseradish peroxidase (1:250). PeroxyGlow A and B reagents (1:1) were added in 100 μL/well aliquots. Chemiluminescence was determined immediately by a Victor 3 multilabel plate reader (Perkin Elmer, Waltham, MA, USA). Data are presented for duplicate samples from one experiment.

### 3.5. DNA Damage (γH2AX) Cytometry

Triplicate cultures of 2 × 10^6^ cells were plated in 10 cm dishes. After indicated treatment, cells were washed, fixed in 70% methanol, and immunostained with a primary antibody γH2AX (mouse anti-human fluorescein isothiocyanate (FITC)-conjugated anti-γH2AX antibody (Millipore, Billerica, MA, USA) used at 1:500 dilution) for 90 min at 4 °C in phosphate buffered saline (PBS) with 2% bovine serum albumin [3]. DNA was stained with propidium iodide at 2 mg/10^6^ cells. Fluorescence was measured by a Coulter EPICS XL-MCL flow cytometer (Beckman Coulter Inc., Miami, FL, USA) using a 488 nm laser. Data were analyzed with WinMDI 2.9 (The Scripps Research Institute, San Diego, CA, USA).

### 3.6. Neutral Single Cell Electrophoresis (Comet) Assays

Triplicate cultures of 2 × 10^5^ cells were plated overnight in 6-well 35-mm plates. The next day, cells were treated as indicated and harvested six hours later by trypsinization followed by scraping for neutral single-cell gel electrophoresis (Comet) assay using a commercial kit (Trevigen, Gaithersburg, MD, USA). Cells were centrifuged (2500× *g* for 5 min), washed in 1X PBS, centrifuged again, and then resuspended in 1X PBS for 1 × 10^5^ cells/mL. Five microlitres of cell suspensions were added to agarose aliquots provided in the kit, mixed, and then pipetted (volume = 25.5 μL) to a 20-well comet slide (Trevigen). Comet slides were cooled at 4 °C for 30 min, immersed in 200 mL chilled lysis buffer in the dark, transferred to chilled 1X neutral electrophoresis buffer at 4 °C for 30 min. Cell/agarose samples were electrophoresed (21 volts) for 60 minutes at 4 °C in neutral buffer (Tris Base/Sodium Acetate/dH_2_0). Microgels were washed, fixed with 70% ethanol for 30 min, dried, and stained with commercial dye for 5 min at 4C (SYBR green, Trevigen). Samples were analyzed for DNA tail moment on a Nikon Eclipse TE2000-S epifluorescence microscope (Tokyo, Japan) at 4× and 10× magnification with computer-based Komet^®^ Assay IV software (Andor™ Technology, Belfast, Ireland). The software calculates the DNA tail moment as the product of the tail length and the total DNA fraction in the tail. The mean number of cells analyzed for DNA tail moment was 29 cells (range 9–50 cells).

### 3.7. Statistical Methods

MANOVA statistics (α = 0.01) using a balanced complete block factorial design were calculated for a global test for differences between IR-ABT-888 and topotecan-ABT-888 responses [28]. The MANOVA statistic tests fitted response vectors for cell survival curves first one parameter at a time, and then, between dose responses. Basically, the MANOVA statistic compares the overall “shape” of non-treated and treated cell survival curves together. The MANOVA statistic was computed using statistical software (α = 0.01, SPSS 18, SPSS Inc., Chicago, IL, USA). PARP activity assays were analyzed by analysis of variance (ANOVA, α = 0.01). Comet assays were analyzed by Wilcoxon rank sum tests (α = 0.01) using the statistical software R [29]. Means and standard errors (SE) are reported.

## 4. Conclusions

Our data show that PARP inhibition protracts DNA double-strand break repair after IR or after topotecan in human cervical cancer cell lines. As anticipated, blockade of PARP by ABT-888 led to increased radiochemotherapy cytotoxicity. The lethal effects of PARP inhibition were more pronounced when cells were challenged to correct topotecan-poisoned replication forks, an observation likely linked to conversion of tolerable single-strand nicks to lethal double-strand breaks [30,31]. This work stands out by demonstrating enhanced cell death by IR or topotecan with PARP inhibition in cells having an augmented chance of repairing DNA readily as a consequence of a high deoxynucleotide output from RNR.

Cervical cancer cells showed sensitivity to ionizing radiation and to topotecan when PARP is inhibited by ABT-888. Whether PARP inhibition during topotecan administration increases therapeutic response rates in women with persistent or recurrent cervical cancer is currently being studied in a phase II clinical trial (Gynecologic Oncology Group protocol #0127W, ClinicalTrials.gov NCT01266447) that was structured after prior work [32].

## Supplementary Files

Supplementary File 1 Supplementary Information (PDF, 420 KB)
