# Supplementary material for: Inhibition of Poly(ADP-Ribose) Polymerase Enhances Radiochemosensitivity in Cancers Proficient in DNA Double-Strand Break Repair
Source: Int J Mol Sci. 2013 Feb 8;14(2):3773–85. doi: 10.3390/ijms14023773 (PMC3588069; doi:10.3390/ijms14023773)
Supplement: Supplementary File 1 — Supplementary Information (PDF, 420 KB) [file ijms-14-03773-s001.pdf]

Supplementary Information

Figure S1. (**a**) Cytometry data for γH2AX induction and resolution over time after radiation (6 Gy) or ABT-888 (5 µM), or the combination are presented as indicated in C33-a cells. A high residual γH2AX signal (48% of cells) persisted at 6 hours after the start of radiation (IR) and ABT-888, indicating the possibility of persistent IR-mediated DNA damage. (**b**) Treatment with ABT-888 significantly protracts γH2AX signal resolution. However, the technique cannot distinguish foci signal from collapse of replication forks or impaired remedy of strand breaks induced by IR. Means and standard errors are shown. (**c**) Photomicrographs of C33-a cells six hours after indicated treatment illustrate γH2AX foci.

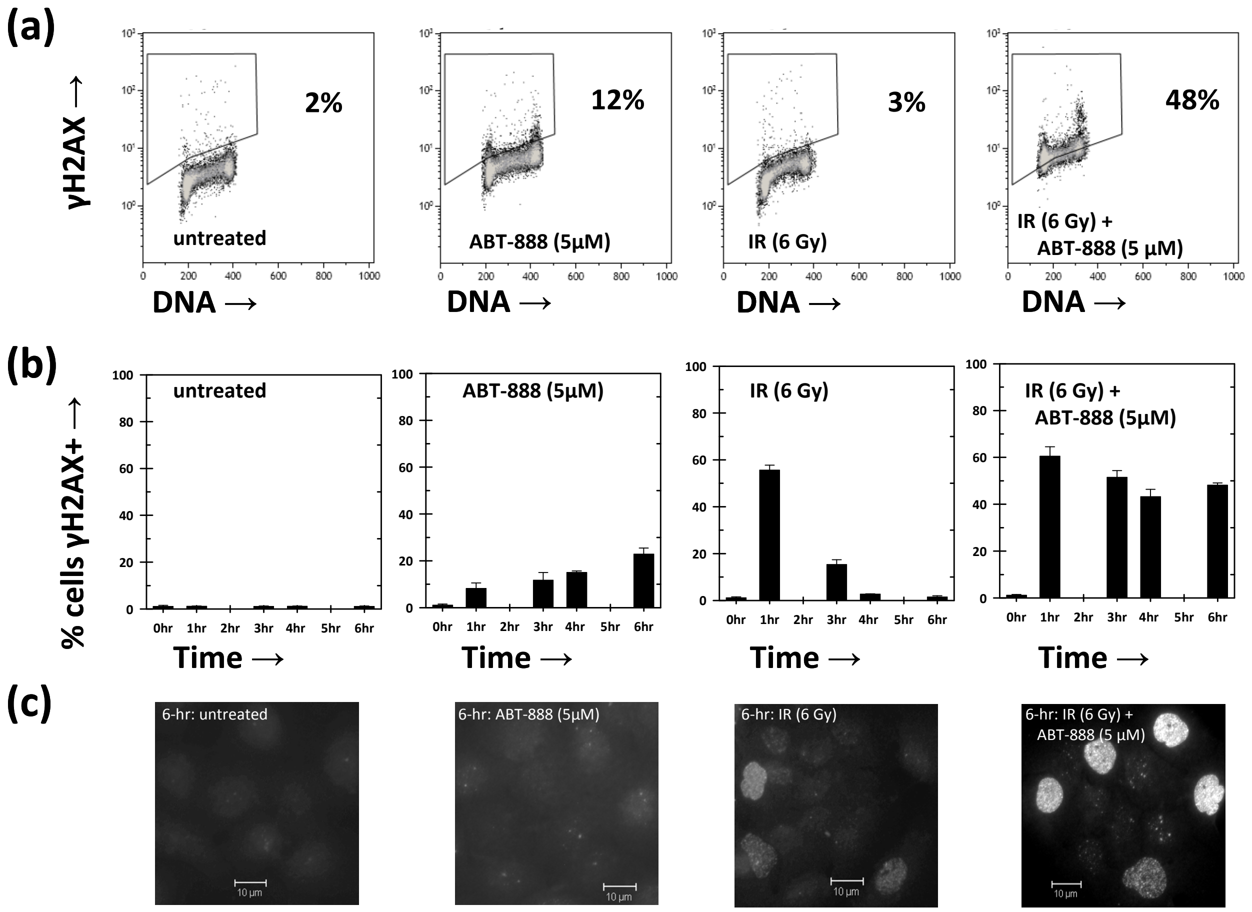

© 2013 by the authors; licensee MDPI, Basel, Switzerland. This article is an open access article distributed under the terms and conditions of the Creative Commons Attribution license (http://creativecommons.org/licenses/by/3.0/).
